# Cortical microstructural changes in schizophrenia spectrum disorders using quantitative T1 mapping

**DOI:** 10.3389/fpsyt.2025.1650055

**Published:** 2025-12-09

**Authors:** George Nader, Kimberly L. Desmond, Matisse Ducharme, Philip Gerretsen, Ariel Graff, Vincenzo De Luca

**Affiliations:** 1 Centre for Addiction and Mental Health (CAMH), Toronto, ON, Canada; 2 Department of Pharmacology & Toxicology, University of Toronto, Toronto, ON, Canada; 3 Institute of Medical Science, Temerty Faculty of Medicine, University of Toronto, Toronto, ON, Canada; 4 Brain Health Imaging Centre, Centre for Addiction & Mental Health (CAMH), Toronto, ON, Canada; 5 Department of Psychiatry, University of Toronto, Toronto, ON, Canada

**Keywords:** schizophrenia, neuropsychiatry, neuroimaging, multimodal imaging, MRI, quantitative T1 mapping

## Abstract

**Introduction:**

Cortical brain changes have long been established in schizophrenia spectrum disorders (SSD), with a particular involvement of frontal and temporal areas. However, most studies to date focused on macrostructural analyses, such as cortical volume, thickness, and surface area. Quantitative T1 imaging (qT1) provides a measure of microstructural tissue properties, corresponding mainly to myelination.

**Methods:**

Fourteen SSD patients and 7 healthy controls were recruited and underwent qT1 imaging using two different acquisition sequences, single and multi-echo qT1.

**Results:**

Compared to controls, SSD patients had a pronounced increase in qT1 values in frontal and temporal areas, while accounting for age and sex. However, this was only detected by the single echo qT1. Additionally, single echo qT1 was negatively modulated by sex in the SSD group, with females having lower qT1 values compared to their male counterparts.

**Discussion:**

These results suggest impaired myelination in the frontal and temporal cortices in SSD. Lastly, we highlight the importance of protocol selection as inter-protocol reliability is still a concern despite the quantitative protocols developed to overcome this limitation.

## Highlights

- Numerous cortical changes have been established in schizophrenia spectrum disorders (SSD), namely in frontotemporal areas.
- We demonstrate decreased cortical myelination evidenced by increased qT1values in SSD.
- Acquisition protocol selection is key as inter-protocol differences significantly affect the findings.

## Introduction

Cortical brain abnormalities have long been recognized in schizophrenia spectrum disorders (SSD), since the early days of neuroimaging research in psychiatry. Changes in the cerebral cortex have been studied using various modalities, most commonly using magnetic resonance imaging (MRI), and have become a key hallmark of the disorder (1). SSD patients consistently display decreased cortical thickness in frontal and temporal areas compared to unaffected healthy controls (2, 3). Additionally, there is evidence of altered cortical gyrification, with mixed reports on the direction and magnitude (4–6). Although these findings significantly expanded our knowledge of the pathophysiology of SSD, they only provide insights into gross-level changes and offer limited clinical utility.

On tissue microstructure level, post-mortem studies have been the main method for studying cortical changes in SSD. The most consistent findings at this level are reduced neuronal cell size and density of inhibitory cortical interneurons (7). Interestingly, immunohistochemistry analyses show a decrease in the number and density of oligodendrocytes in frontal and parietal cortices (Boradman Area 10 and 39) (8–10). This was further affirmed by a decrease in mRNA expression levels of myelin-related proteins in the anterior cingulate cortex (11). Although demyelination has been mostly recognized in white matter, these findings suggest its presence in the cortex as well in SSD. However, post-mortem analyses are limited by the small sample size and the inability to control for confounding variables. *In vivo* research in this area is also scarce, mainly due to the recency of non-invasive myelin mapping imaging tools.

Flynn et al. utilized transverse relaxation (T2) mapping as a proxy of myelin content in schizophrenia (12). Moreover, recent studies explored cortex-wide myelin mapping *in vivo* using T1w/T2w MRI in first-episode psychotic patients (13, 14). However, this area remains largely unexplored outside the white matter in chronic SSD patients. Additionally, other quantitative mapping techniques, such as longitudinal relaxation time or quantitative T1 (qT1), were shown to be superior to other myelin mapping techniques in terms of inter and intra-subject variability, as well as contrast-to-noise ratio (15). QT1 values are influenced by numerous factors, such as myelin composition, cellular density, iron concentration, free and bound water (16). Although the relative contribution of each factor is not fully understood, myelin appears to be the primary contributor (17–19).

In previous work, we found evidence of elevated qT1 values in SSD patients in several subcortical areas (20). Additionally, we assessed the inter-protocol sensitivity and reliability since qT1 mapping was performed with two sequences: single-echo and multi-echo fast spoiled gradient echo (fSPGR). The latter sequence is especially of interest due to its ability to generate multimodal images of qT1, T1-weighted, T2*, quantitative susceptibility mapping (QSM), and susceptibility weighted imaging (SWI) (4, 21–23). Since more sophisticated neuroimaging approaches are being utilized in neuropsychiatry, multimodal sequences present exciting avenues because they eliminate the need to acquire separate scans for each metric. This is of great importance in psychiatric populations, where clinical instability and agitation may lead to exclusion from projects with longer scans. Additionally, multimodal imaging minimizes preprocessing and co-registration of several images, which could mitigate the reproducibility problem common in neuroimaging (24, 25). However, it is important to carefully weigh the costs and benefits of both sequences, since different acquisition protocols may incur different sensitivities to microstructural changes or to several factors, such as age, sex, and antipsychotic medications. Thus, we investigated cortical qT1 changes in SSD patients relative to healthy controls, and assessed the inter-protocol variation of the two aforementioned sequences.

## Methods

### Participants and recruitment

Fourteen participants with schizophrenia spectrum disorders (SSD) and 7 healthy controls (HC) were recruited at the Centre for Addiction & Mental Health (CAMH), in Toronto. The SSD diagnosis was ascertained with the Mini-International Neuropsychiatric Interview (MINI) for the DSM-5 (26). The HC group were free of any primary psychosis, and the SSD group were all receiving antipsychotic medications. Exclusion criteria for both groups included: a history of traumatic brain injury, substance-induced psychosis, and a diagnosis of intellectual disability or major neurological disorders. In the SSD group, two participants had a diagnosis of schizoaffective disorder, while all the others were diagnosed with schizophrenia. Notably, the patients’ group was significantly older; however, the two were comparable in sex and ethnicity. Demographic breakdown of participants is presented in **Table 1**.

**Table 1:** Demographic breakdown of study participants.

Demographic Variable	SD (n=14)	HC (n=7)	P-value
Age (years)	47 ± 14	28 ± 9.30	0.0009
% Male	57	57	0.875
% Caucasian	46.67	14.29	0.117
Chlorpromazine equivalents (CPZe)	548 ± 320	–	–

### MRI acquisition parameters

All scans were acquired using a 3T GE scanner with an integrated radiofrequency pulse (RF) body coil and an 8-channel receiving head coil (GE Healthcare, Waukesha, WI). Two protocols were employed for quantitative T1 (qT1) mapping: 1) Sagittal, single spoiled gradient echo (SPGR) with two flip angles (α = 3° and 14°), TE = 4.948 ms, and voxel size = 1x1x1mm^3^. This protocol is referred to as single-echo (SE-qT1) in the manuscript. 2) Axial fast SPGR with two flip angles (α = 3° and 24°), 5 echoes (TE = 3.36 ms, echo spacing = 4.42 ms, TR = 28.5 ms), and a voxel size = 0.5x0.5x2mm^3^. This protocol is referred to as multi-echo (ME-qT1) in the manuscript. To correct for B1 field inhomogeneity, the fast spin echo–double angle method (FSE-DAM) was employed as described in Samson et al. (27). Briefly, two multi-slices sagittal FSE images (TR = 15 s, Effective-TE=14 ms, echo train length = 8) were acquired with two excitation flip angles (α = 60° and 120°). Lastly, T1-weighted images were acquired for surface segmentation, using standard sagittal T1 BRAVO sequence: FA = 8°, single echo (TR = 6.868 ms, TE = 3.016 ms, TI = 650 ms), and voxel size: 0.9x0.9x0.9mm^3^.

### T1 maps generation and processing

T1 maps were generated as previously described in Nader et al. (20). For ME-qT1, the five echoes were averaged for each flip angle. qT1 images were aligned to their halfway space, and B1 correction factors were calculated and scaled by a scanner specific constant to create the B1 map (28). Voxel-wise flip angle correction was then performed and the T1 relaxation time was then fit from the spoiled gradient signal equation.

To obtain surface segmentation, T1-weighted images were processed using the FreeSurfer 7.1.1 recon-all command (29), and parcellated using the Desikan-Killiany atlas (30). Quality control inspection was performed, and only minimal editing was required for intensity normalization and pial surface error correction. T1 maps for each subject were then projected to the middle of the cortical surface using FreeSurfer’s *mri_vol2urf*, with – *projfrac 0.5.*

### Statistical analysis

First, a correlation analysis was performed for each ROI between SE-qT1 and ME-qT1. All of the following analyses were performed for both protocols. To visualize case-control differences, surface maps were averaged into FreeSurfer’s *fsaverage* space using *mris_preproc* and displayed after vertex-wise analysis. For ROI-specific case-control differences, average qT1 values in each ROI were extracted, and the differences were tested with the general linear model function *lm* in R, while adjusting for age and sex (31). Lastly, a covariate analysis was performed in the SSD group to test the sensitivity of each protocol to age, sex, and chlorpromazine equivalents (CPZe), while accounting for the other two covariates. The false-discovery rate (FDR) - Benjamini-Hochberg method was employed to correct for multiple comparisons, and all the analyses were performed separately for the right and left hemispheres.

## Results

### Inter-protocol qT1 correlation

**Figure 1:**
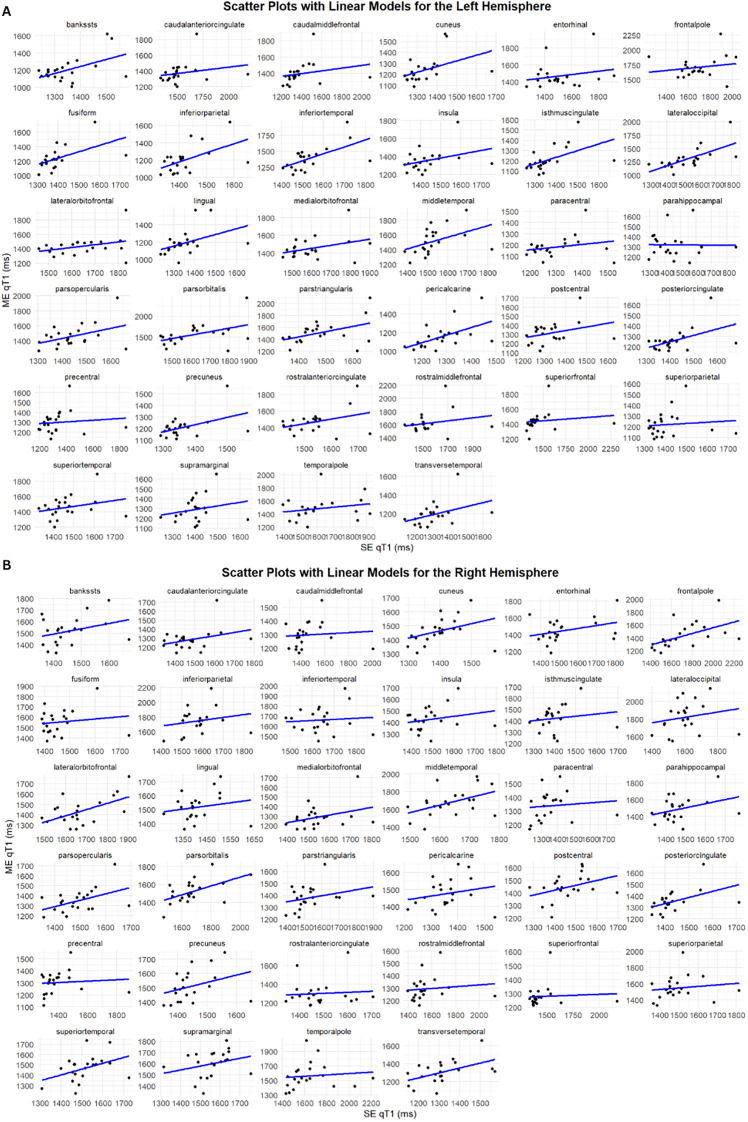
Variable correlation between SE-qT1 & ME-qT1 in various ROIs. Each datapoint represents the values of a single subject. The scatter plot shows a linear line of best fit (n = 21). SE-qT1: single-echo quantitative T1, ME-qT1: multi-echo quantitative T1.

First, the correlation between SE-qT1 and ME-qT1 values in each ROI was tested. In the left hemisphere, only 6/34 cortices (isthmus cingulate, fusiform, middle temporal, cuneus, lateral occipital, and pericalcarine) were significantly correlated after correcting for multiple comparisons (**Figure 1A**). Surprisingly, no significant correlations were found in the right hemisphere after correcting for multiple comparisons (**Figure 1B**).

### Cortical microstructural alterations in SSD

**Figure 2:**
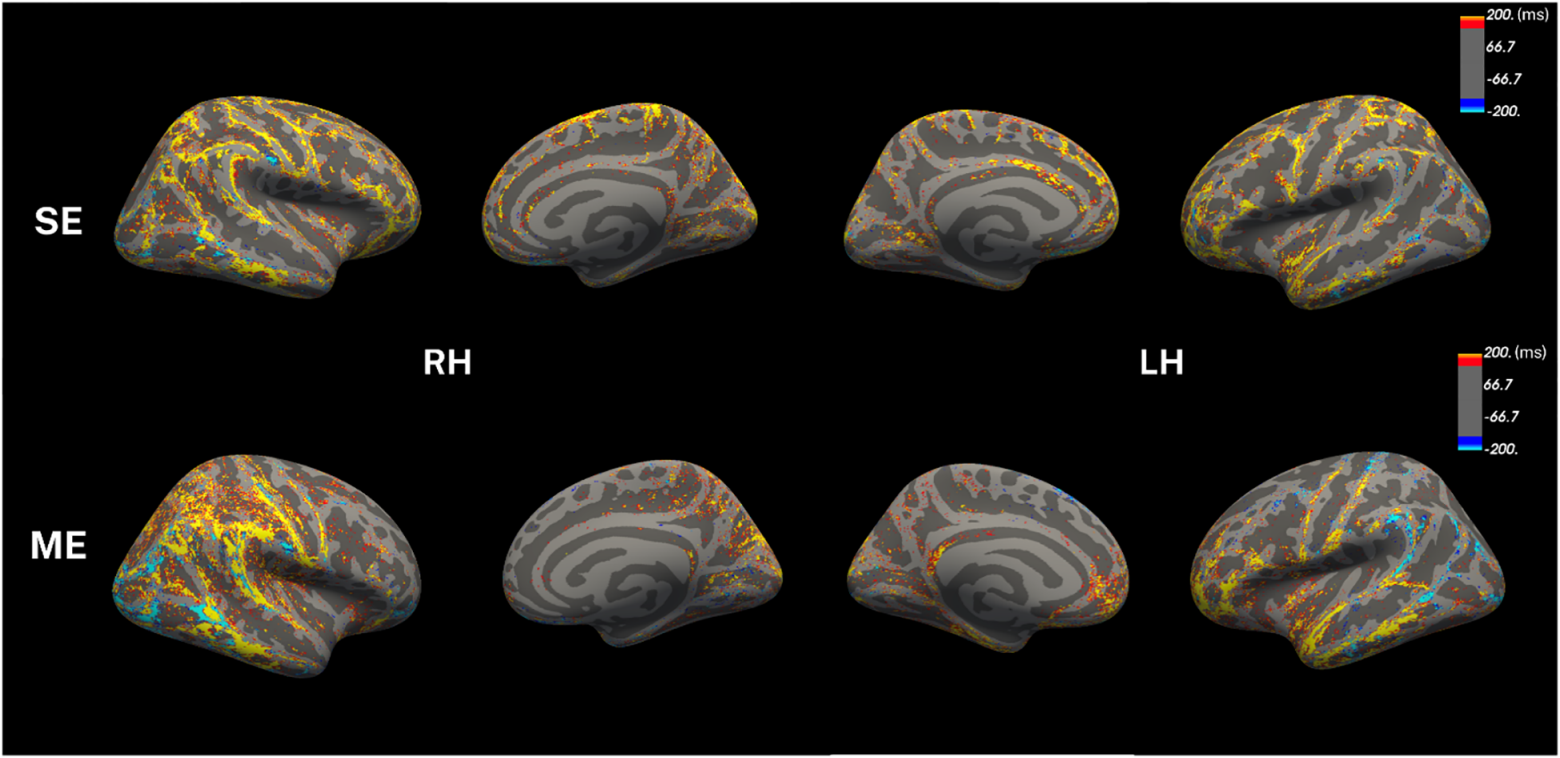
qT1 differences in SSD compared to healthy controls. Vertex-wise analysis (SSD – HC) was conducted for visualization only, and the color bar shows values in milliseconds (ms). The same analysis was repeated in both protocols (n = 14 for SSD, n = 7 for HC).

To visualize the differences in qT1 values between SSD and healthy controls (HC), qT1 maps were averaged onto a common surface and vertex-wise difference was calculated (**Figure 2**). Overall, both protocols show higher qT1 values in SSD compared to HC, especially in left frontal and temporal lobes, as well as the right temporoparietal areas (**Figure 2**). There were few parietal and occipital regions with decreased qT1 values in SSD patients, however this appeared localized (**Figure 2**). It should be noted, however, that the ME-qT1 showed larger, and more diffuse changes compared to SE-qT1, as highlighted in the widespread right temporoparietal areas in **Figure 2**.

**Figure 3:**
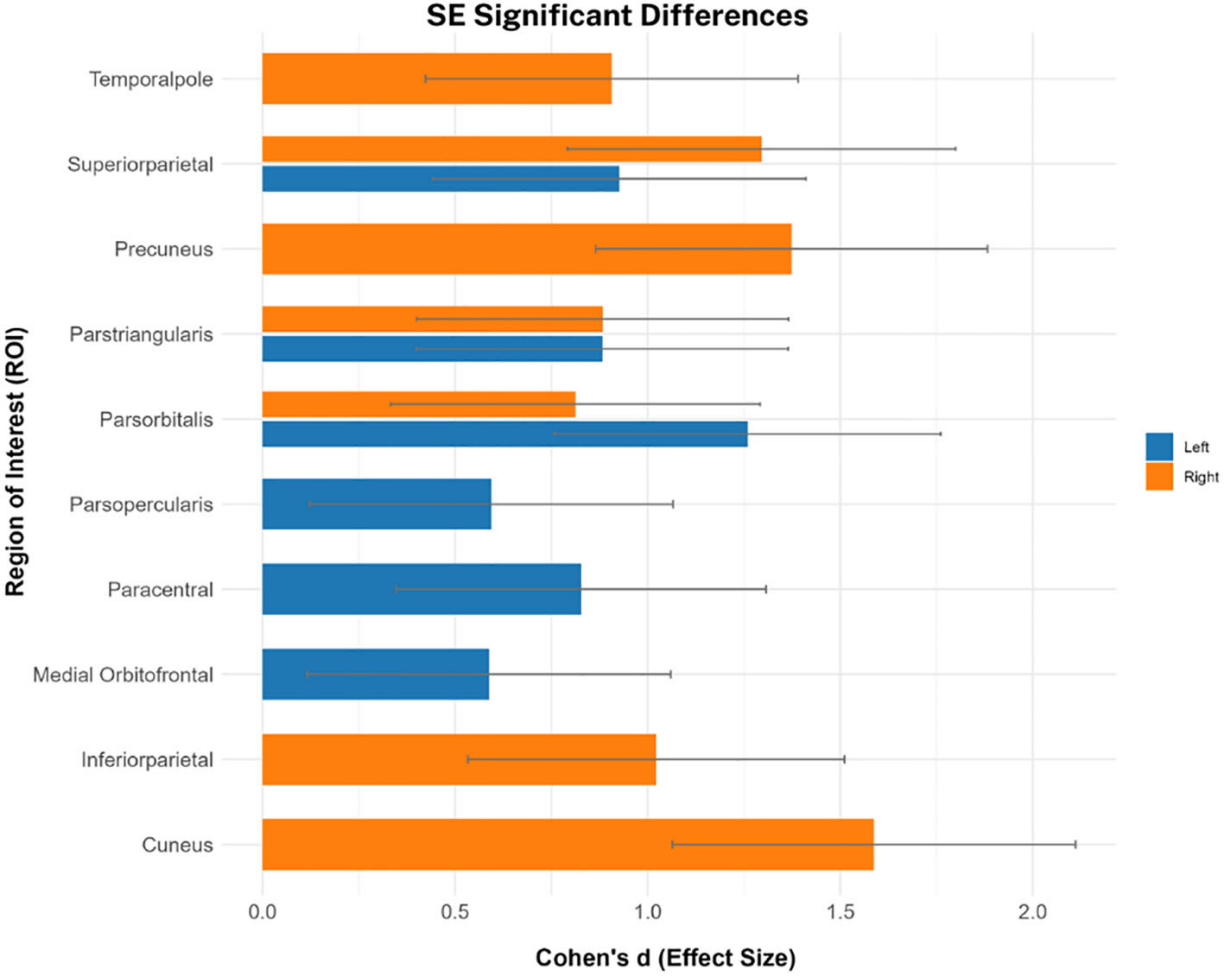
ROI-specific qT1 significant differences in SSD vs. healthy controls. Bar plot showing only statistically significant effect sizes from SE-qT1 after adjusting for age and sex and correcting for multiple comparisons (FDR-adjusted p < 0.05). Error bars showing the standard error (n = 14 for SSD, n = 7 for HC).

**Figure 4:**
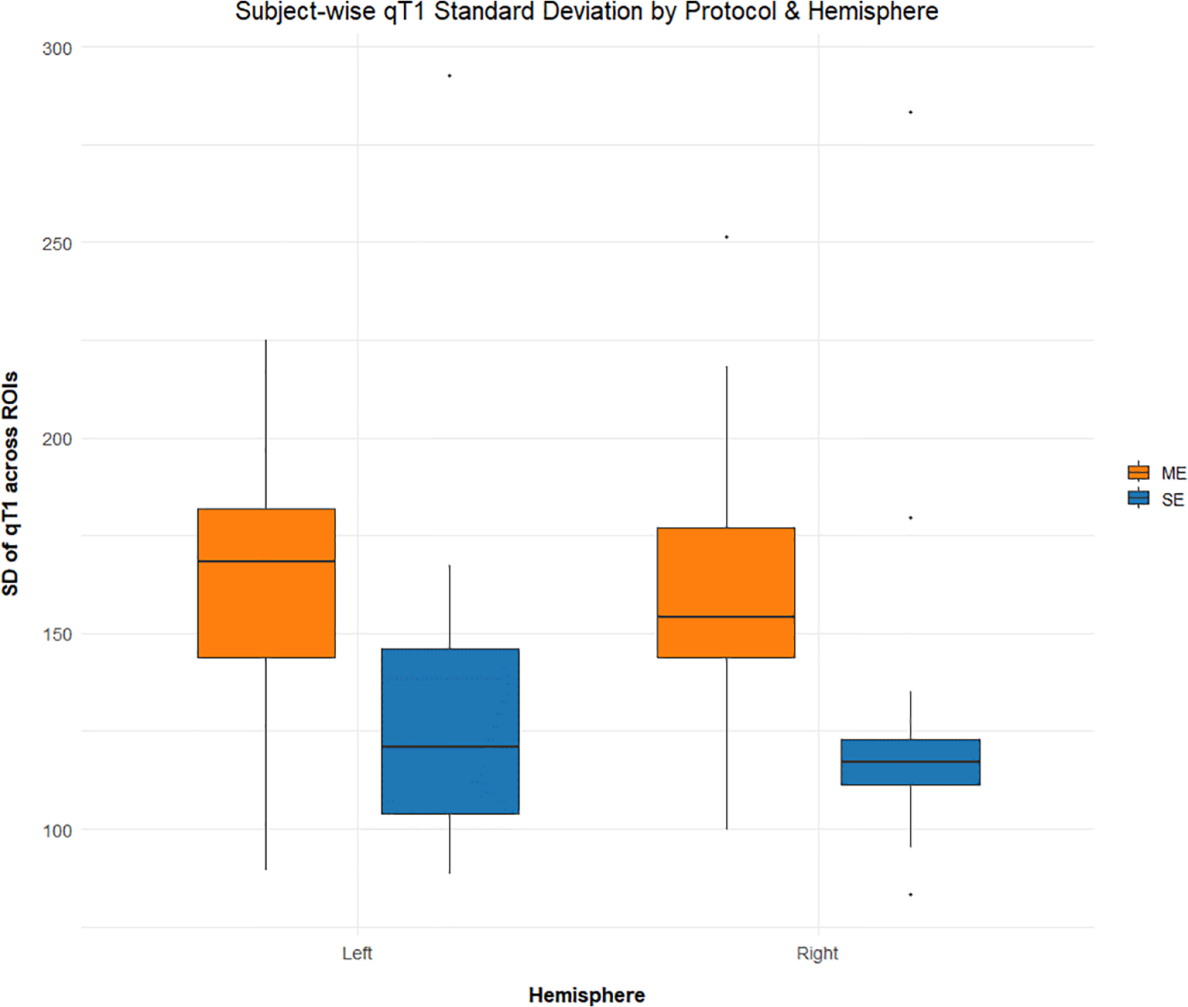
Average qT1 standard deviation by hemisphere, for each protocol. Boxplot showing the average standard deviation for the pooled sample across all ROIs (n = 14. for SSD, n = 7 for HC).

To further investigate qT1 changes in each ROI, average values were compared per parcel between SSD and HC. Surprisingly, unadjusted case-control differences were not significant in any ROI after correcting for multiple comparisons. However, after correcting for age and sex, only SE-qT1 showed significantly elevated qT1 values in SSD vs. HC (**Figure 3**). These differences spanned all four lobes, and effect sizes ranged from 0.59 in the left medial orbitofrontal to 1.59 in the right cuneus (**Figure 3**, **Table 2**). There was no case-control significant difference detected by the ME-qT1 acquisition protocol. Lastly, it should be noted that the standard deviation in qT1 values was higher in the ME-qT1 dataset, compared to SE-qT1 (**Figure 4**).

**Table 2:** Case-control differences for SE-qT1.

Region of Interest	SSD – HC mean difference (ms) (mean ± standard error)	Effect size (Cohen’s D ± standard error)	FDR-adjusted p-value
Right hemisphere			
*R-temporal pole*	171.05 ± 87.30	0.9 ± 0.48	0.037
*R-superior parietal gyrus*	120.54 ± 43.06	1.3 ± 0.50	0.021
*R-inferior parietal gyrus*	80.76 ± 36.58	1.02 ± 0.49	0.037
*R-pars triangularis*	95.31 ± 49.95	0.88 ± 0.48	0.037
*R-pars orbitalis*	110.8 ± 63.16	0.81 ± 0.48	0.037
*R-precuneus gyrus*	81.76 ± 27.55	1.37 ± 0.51	0.021
*R-cuneus gyrus*	85.90 ± 25.05	1.59 ± 0.52	0.021
Left hemisphere			
*L-superior parietal gyrus*	88.93 ± 44.45	0.93 ± 0.48	0.049
*L-pars triangulairs*	82.00 ± 43.03	0.88 ± 0.48	0.049
*L-pars orbitalis*	158.38 ± 58.21	1.26 ± 0.50	0.049
*L-pars opercularis*	56.23 ± 43.88	0.59 ± 0.47	0.049
*L-paracentral gyrus*	73.79 ± 41.29	0.83 ± 0.48	0.049
*L-medial orbitofrontal gyrus*	72.12 ± 56.78	0.59 ± 0.47	0.049

### Covariates analysis of qT1 values in SSD

**Figure 5:**
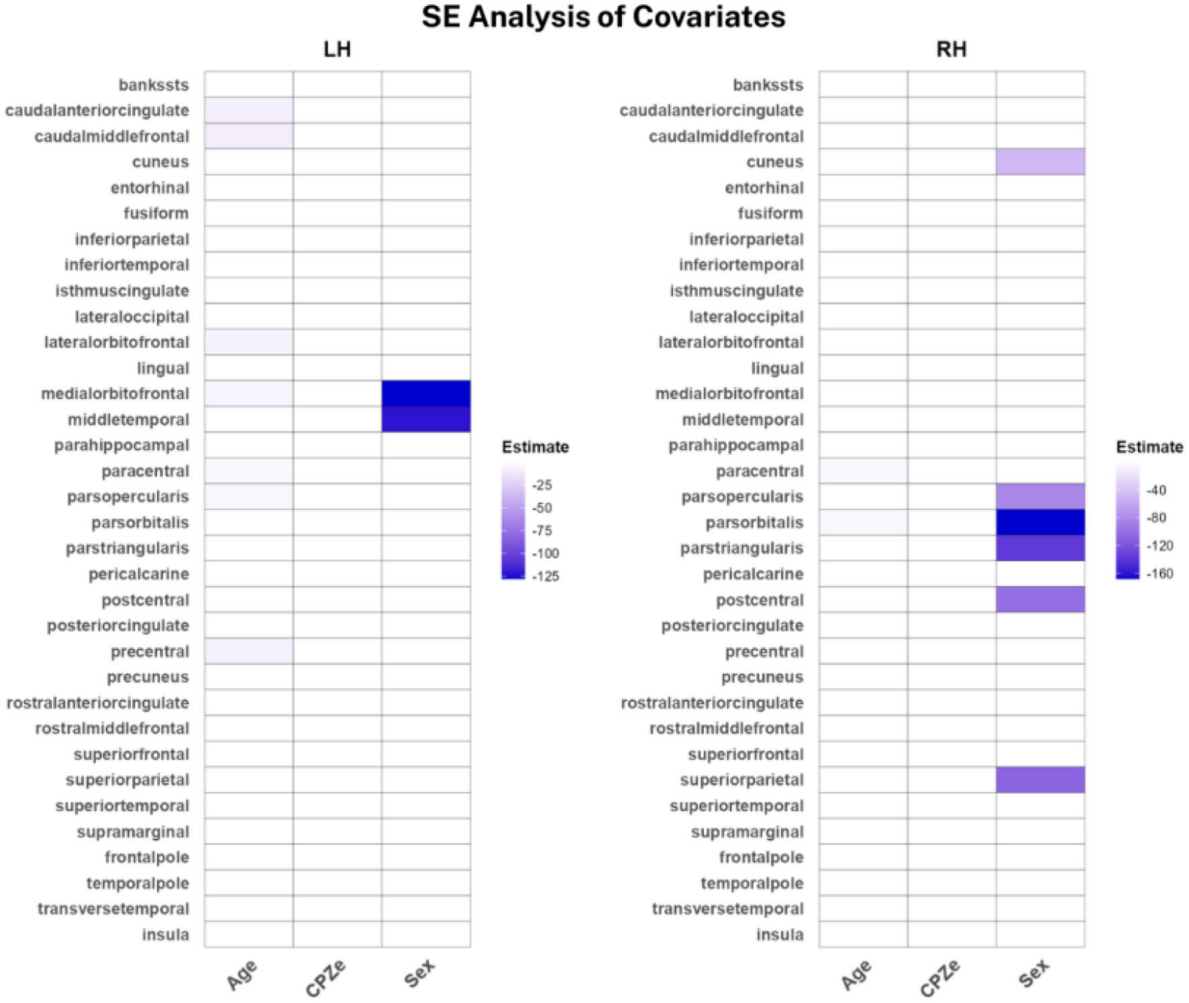
Heat maps of significant covariates in SE-qT1. Colored boxes indicate a significant covariation. Color bar showing linear regression coefficient. Only significant modulations are shown, and the rest are noted as zero for better visualization (n = 14).

**Figure 6:**
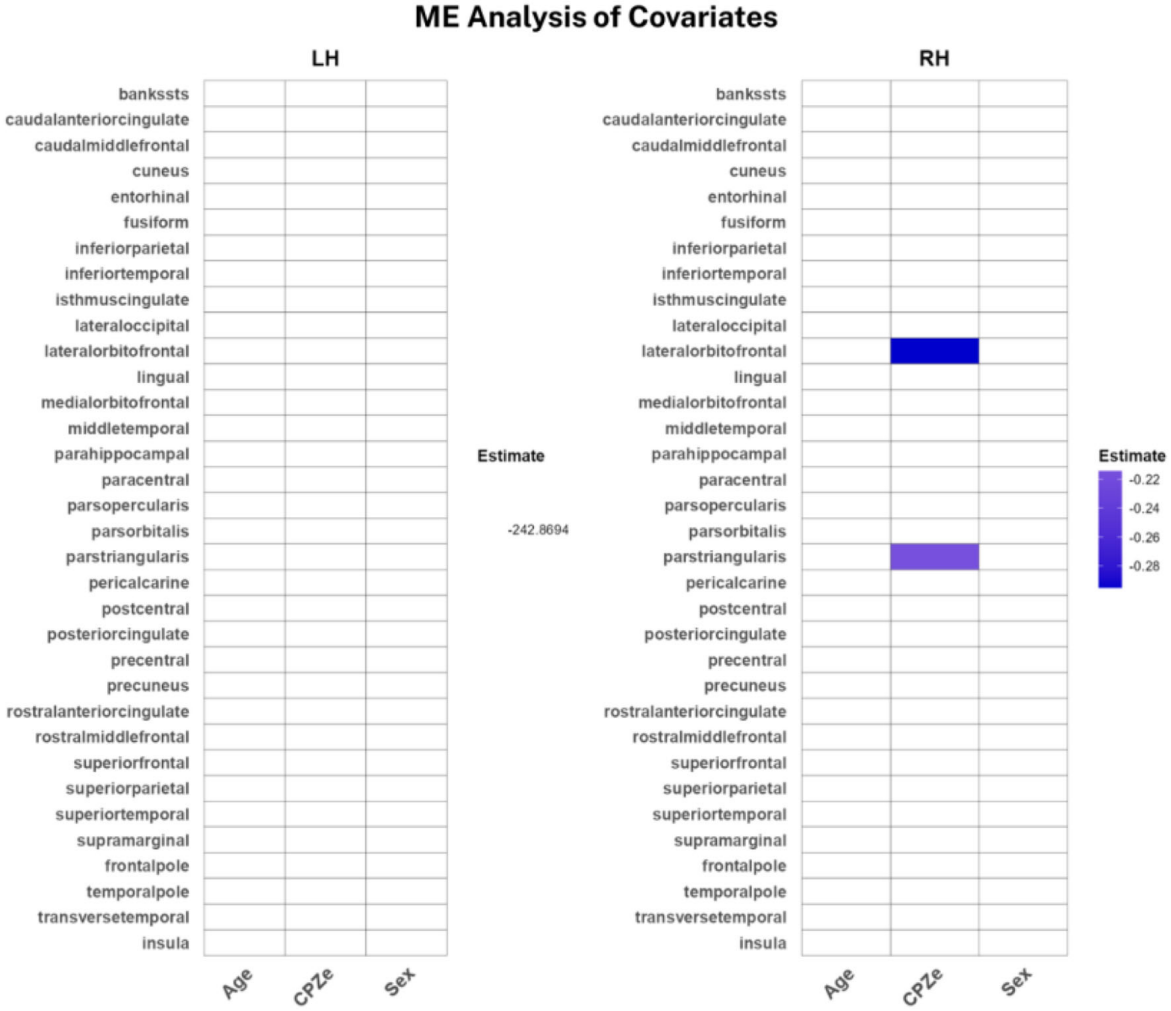
Heat maps of significant covariates in ME-qT1. Colored boxes indicate a significant covariation. Color bar showing linear regression coefficient. Only significant modulations are shown, and the rest are noted as zero for better visualization (n = 14).

Lastly, we investigated each protocol’s susceptibility to variation in age, sex, and CPZe in the SSD sample. For SE-qT1, females appeared to have lower qT1 values compared to males, while adjusting for age and CPZe. The strongest effects were localized in the bilateral frontal and temporal areas, with milder effects in the parietal and occipital areas (**Figure 5**). As for the ME-qT1, values were negatively modulated by CPZe, while adjusting for age and sex in the right pars triangularis and lateral orbitofrontal cortices (**Figure 6**). No significant effects were found in the left hemisphere for ME-qT1.

## Discussion

In this work, we present a novel analysis of cortical myelination changes in SSD using quantitative T1 imaging. Furthermore, we also assessed the inter-sequence reliability of two different acquisition methods: standard single echo qT1 imaging (SE-qT1) and fSPGR multi-echo qT1 imaging (ME-qT1). This analysis is the first of its kind, as all previous reports were conducted using different imaging sequences and in drug-naïve first-episode/SSD patients.

First, the two protocols appeared to be weakly to moderately correlated (**Figures 1A, B**), however, this only reached statistical significance in six parcels in the left hemisphere, and none in the right. Considering that the two qT1 maps were projected on the same cortical surface, discordance between the two is likely to be due to the T1 map itself rather than cortical surface differences. Although in previous work we reported significantly correlated values in the cerebral cortex, there are several reasons that could explain this discrepancy (20). First, the total cerebral cortex was averaged and considered in the previous analysis without assessing individual parcels as we present here. Additionally, our surface-based approach here contrasts with the voxel-based one used in the previous manuscript. Although the two protocols are significantly correlated in the cortex on average, this correlation breaks down as the volume of the regions decreases, with a corresponding increase in uncertainty.

Furthermore, there was a significant increase in qT1 values in SSD, however, only measured by SE-qT1. This increase in qT1 values generally indicates pathological changes, such as neuroinflammation, reduced cellular/synaptic density, increased iron deposition, and, primarily, less myelination. First, medium to large effect sizes were observed in the inferior frontal areas (L/R-pars orbitalis, L-pars triangularis, L-pars opercularis, and L-medial orbitofrontal) (**Figure 3**, **Table 2**). These changes are consistent with post-mortem evidence of dysfunctional myelination in frontal areas (8, 9). Additionally, it is in line with recent meta-analyses suggesting significantly decreased cortical thickness and grey matter volume in the inferior temporal gyrus in SSD (32, 33). Although cortical thickness and qT1 changes are two distinct phenomena, the two may still be interconnected. At the surface level, these changes indicate the susceptibility of these brain regions to changes in SSD. More specifically, cortical thinning in early adolescence is associated with genetic variance in dendritic cytoarchitecture and myelin-related genes (34). These genetic alterations were also implicated in psychiatric illnesses.

In the only other investigation of cortical myelination in first-episode psychosis, reduced myelination in the deeper layer of the inferior frontal cortex was demonstrated, however, not at a statistically significant level (13). This does not contradict our findings as frontal changes become more pronounced with illness progression, which is the case in our sample. For instance, Zhao and colleagues found reduced pars orbitalis cortical thickness in chronic schizophrenia but not in first-episode patients (33). Furthermore, there were similar changes in the temporal pole, superior parietal, cuneus, and precuneus gyri (**Figure 3**), all of which are supported by gross changes in grey matter volume and surface area in the literature (6, 33, 35, 36).

On the other hand, there were no significant changes detected by ME-qT1, although it had similar effect sizes. This could be explained by the larger variability in its values, with a larger standard deviation in the left and right hemispheres (**Figure 4**). This may have prevented statistical significance achievement and indicates that this qT1 derivation method from multi-echo sequences may not be as accurate as the single-echo qT1. A similar trend was seen in our previous work, where only SE-qT1 detected changes in the hippocampus (20). Although multi-echo acquisition allows for multimodal imaging, it may compromise the sensitivity to microstructural changes due to increased variability from averaging multiple echoes (**Figure 4**). Specifically, later ME echoes are known to be increasingly affected by T2* decay, which is modulated by iron and free water content, along with other microstructural factors (37). Thus, the signal-to-noise ratio may decrease with the additive accumulation of noise from each echo while the signal, at best, remains stable. Selecting the first two echoes or weighting each echo by echo time (TE) may help reduce this variability in future investigations.

Lastly, the covariates analysis indicated that only SE-qT1 was sensitive to sex, with females having lower qT1 values in several left and right cortices while accounting for age and CPZe (**Figure 5**). This is consistent with our previous subcortical investigation. Moreover, qT1 values fluctuate with age; however, only SE-qT1 showed a very subtle decrease with age (**Figure 5**). Although this is contrary to the expected trend, the small sample size and relatively narrow age range may underlie this discrepancy (38). Additionally, it is not clear whether the qT1 values in SSD follow a similar trend with age as in healthy subjects. Interestingly, ME-qT1 showed lower qT1 values with increased CPZe, possibly indicating increased myelination or cellular density (**Figure 6**). This may be explained by the fact that over 70% of participants were on second generation antipsychotics, which are generally thought to be less detrimental to brain structure than first generation ones (3, 39).

Altogether, our work suggests abnormal myelination in several brain areas of SSD patients, and that these changes are modulated by factors such as antipsychotic medications. These findings further contribute to our understanding of the pathophysiology of SSD and provide promising avenues for clinical utility. For instance, Wei et al. found that myelination in certain parietal and insular areas correlates with excitement and depression scores (13). Considering the differences between our chronic SSD sample and the participants in Wei’s study, it is also plausible that qT1 changes dynamically fluctuate throughout the illness and therefore can be utilized to track symptomatic and functional prognosis. Notably, qT1 imaging also has excellent scan-rescan and inter-scanner reliability, making it highly suitable for multi-centric studies (40). This paves the way for future longitudinal investigations with larger samples that may reveal illness and medication-related effects on microstructure. By utilizing machine learning and advanced data analytics, such sensitive imaging methods may have diagnostic and prognostic utilities in the future.

There were several strengths and limitations to this work. To the best of our knowledge, we are the first to report on cortical-specific qT1 alterations in SSD and contrast two different methods of acquisition. Additionally, the use of the same T1-weighted surface for the qT1 map projection eliminates sources of error that may arise from using lower-resolution T1-weighted images derived separately from each quantitative sequence. As *in vivo* quantitative MRI is only recently being utilized in psychiatric research, the findings may well enhance our understanding of the pathophysiology of the illness. However, the small sample size severely limits our ability to draw firm conclusions. Rather, this preliminary work optimizes our tools for qT1 mapping in SSD and provides the framework to do so in larger and multi-centric studies. Although segmentation quality control was performed, partial contributions from pial surfaces and white matter cannot be ruled out. Finally, age-matching was not feasible due to our small sample size.

## Conclusion

In conclusion, we show decreased myelination and altered cortical microstructure in schizophrenia spectrum disorders compared to healthy controls, evidenced by increased qT1 values. Additionally, we also demonstrate that single echo qT1 acquisition may be more sensitive than multi-echo qT1, highlighting the importance of sequence selection. However, research in this area remains limited, and future studies that investigate the relationship between tqT1 mapping and illness outcomes will be crucial to understanding the pathology of schizophrenia.

## Data Availability

The raw data supporting the conclusions of this article will be made available by the authors, without undue reservation.
